# Comprehensive high-throughput meta-analysis of differentially expressed microRNAs in transcriptomic datasets reveals significant disruption of MAPK/JNK signal transduction pathway in Adult T-cell leukemia/lymphoma

**DOI:** 10.1186/s13027-021-00390-3

**Published:** 2021-06-29

**Authors:** Shahrzad Shadabi, Nargess Delrish, Mehdi Norouzi, Maryam Ehteshami, Fariba Habibian-Sezavar, Samira Pourrezaei, Mobina Madihi, Mohammadreza Ostadali, Foruhar Akhgar, Ali Shayeghpour, Cobra Razavi Pashabayg, Sepehr Aghajanian, Sayed-Hamidreza Mozhgani, Seyed-Mohammad Jazayeri

**Affiliations:** 1 Student Research Committee, Alborz University of Medical Sciences, Karaj, Iran; 2 Department of Virology, School of Public Health, Tehran University of Medical Sciences, Tehran, Iran; 3 Research Center for Clinical Virology, Tehran University of Medical Sciences, Tehran, Iran; 4 Blood Transfusion Research Center, High Institute for Research & Education in Transfusion Medicine, Tehran, Iran; 5 Hematology-Oncology and Stem Cell Transplantation Research Center, Shariati Hospital Tehran University of Medical Sciences, Tehran, Iran; 6 Blood Transfusion Research Center, High Institute for Research & Education in Transfusion Medicine, Tehran, Iran; 7 Non-communicable Diseases Research Center, Alborz University of Medical Sciences, Karaj, Iran; 8 Department of Microbiology, School of Medicine, Alborz University of Medical Sciences, Karaj, Iran

**Keywords:** HTLV-1, Adult T-cell leukemia/lymphoma, Systems biology, Gene expression analysis, JNK pathway

## Abstract

**Background:**

Human T-lymphotropic virus 1 (HTLV-1) infection may lead to the development of Adult T-cell leukemia/lymphoma (ATLL). To further elucidate the pathophysiology of this aggressive CD4+ T-cell malignancy, we have performed an integrated systems biology approach to analyze previous transcriptome datasets focusing on differentially expressed miRNAs (DEMs) in peripheral blood of ATLL patients.

**Methods:**

Datasets GSE28626, GSE31629, GSE11577 were used to identify ATLL-specific DEM signatures. The target genes of each identified miRNA were obtained to construct a protein-protein interactions network using STRING database. The target gene hubs were subjected to further analysis to demonstrate significantly enriched gene ontology terms and signaling pathways. Quantitative reverse transcription Polymerase Chain Reaction (RTqPCR) was performed on major genes in certain pathways identified by network analysis to highlight gene expression alterations.

**Results:**

High-throughput in silico analysis revealed 9 DEMs *hsa-let-7a, hsa-let-7g, hsa-mir-181b, hsa-mir-26b, hsa-mir-30c, hsa-mir-186, hsa-mir-10a, hsa-mir-30b, and hsa-let-7f* between ATLL patients and healthy donors. Further analysis revealed the first 5 of DEMs were directly associated with previously identified pathways in the pathogenesis of HTLV-1. Network analysis demonstrated the involvement of target gene hubs in several signaling cascades, mainly in the MAPK pathway. RT-qPCR on human ATLL samples showed significant upregulation of *EVI1*, *MKP1*, *PTPRR,* and JNK gene vs healthy donors in MAPK/JNK pathway.

**Discussion:**

The results highlighted the functional impact of a subset dysregulated microRNAs in ATLL on cellular gene expression and signal transduction pathways. Further studies are needed to identify novel biomarkers to obtain a comprehensive mapping of deregulated biological pathways in ATLL.

## Introduction

Human T lymphotropic virus 1 (HTLV-1) is a single-stranded positive-strand RNA virus, which primarily infects CD4+ T-cells in humans [1]. At least 5-10 million individuals have been virus carriers around the globe in several endemic foci including southern Japan and the Caribbean [2]. A subpopulation of individuals infected with HTLV-1 (6% of male and of 2% female subjects) develop Adult T-cell leukemia/lymphoma (ATLL) after a long latency period of 4 to 6 decades [3, 4]. ATLL is a malignant T-cell neoplasm characterized by pleomorphic leukemic cells with hyper segmented nuclei, which are immunophenotypically comparable to regulatory T-cells [5]. This aggressive peripheral T-cell malignancy is associated with a poor prognosis and numerous clinical complications, such as hypercalcemia and immunodeficiency [4]. Since the initial description of ATLL with aggressive subtypes (acute, lymphomatous, and chronic with unfavorable prognostic factors) having a survival rate of less than one year and despite numerous modalities and therapeutic approaches, the median survival rate has not been improved significantly [4, 6]. The oncogenic properties of the viral products are substantiated through countless experiments [7, 8]. However, the low prevalence of ATLL among HTLV-1 carriers and the long latency period suggests that factors such as host genetic susceptibility and environmental factors may influence the development of the disease.

The pX region of HTLV-1 genome encodes two important regulatory proteins HBZ and Tax [9]. The substantial role of Tax and HBZ in HTLV-1 leukemogenesis is evident in the induction of T-cell lymphoma by transgenic expression of each of these transcripts in animal models [10, 11]. Interactions of HTLV-1 Tax and HBZ proteins with host machinery lead to diverse changes in cellular behavior through alterations of signal transduction pathways and gene expression marked by modulation of NF-κB, MAPK, AP-1, JAK/STAT, mTOR, IRFs, TGF-β, and p53 signaling pathways in HTLV-1 infected cells [12, 13]. The complex interactions of Tax and HBZ with host cellular pathways lead to increased proliferation and immune escape of the infected T-cells [14]. The leukemogenic effects of HTLV-1 transcripts are partially explained by their introduction of DNA instability and impairing DNA damage repair, which are signified by various mutations in immortalized ATLL cells [9, 15]. Many of these mutations also converge on pathways already dysregulated by direct interaction of Tax [16], which suggests their role in compensation of loss of Tax expression in the chronic infected cells and their capacity to progress and maintain the leukemic state during the later stages of infection [13, 17]. The higher rate of proliferation and greater survival advantage of HTLV-1 infected cells harboring Tax-mimicking mutations and negative selection of other clones in ATLL may explain the narrower and more uniform clonality of ATLL CD4+ T-cells compared to those of HTLV-1 associated myelopathy/Tropic spastic paraparesis (HAM/TSP) patients and asymptomatic carriers [18].

The ATLL specific genomic signature is not only reflected by the pre-transcriptional and transcriptional modification of gene expression. Infection with HTLV-1 virus and development of ATLL has been associated with significant dysregulation of microRNA (miRNAs) transcriptome in host cells, despite HTLV-1 not having any genome-encoded miRNAs [14, 19–21]. Indeed, the global downregulation of miRNAs in HTLV-1 infected cells by EZH2-induced trimethylated H3K27 histone (H3K27me3) has been cited as a crucial step in the development of ATLL [22]. Furthermore, the expression of DICER1 gene is also reduced in both HAM/TSP [12] and ATLL patients [23] further contributing to lower mature miRNAs in HTLV-1 infected cells. This transcriptomic profile is associated with a poor prognosis in several other malignancies such as hepatocellular carcinoma [24] and invasive breast carcinoma [25]. HTLV-1 is also associated with deregulation of numerous single miRNAs in infected cells which interfere with various biological processes, especially cell cycle regulation [26]. Analysis of these specific gene regulation signatures may reveal key molecular targets for novel treatment modalities in targeted cancer therapies for ATLL.

Here we have conducted an integrated approach to analyze gene expression profiling studies to elucidate the anomalies in miRNA gene regulation system in infected cells through high-throughput analysis of previous transcriptomic datasets in the literature. The enriched pathways and highlighted genes in this study revealed novel disruptions in cell signaling cascades which were then confirmed by real time-PCR.

## Materials and Methods

### Database search and inclusion of eligible datasets

**Fig. 1:**
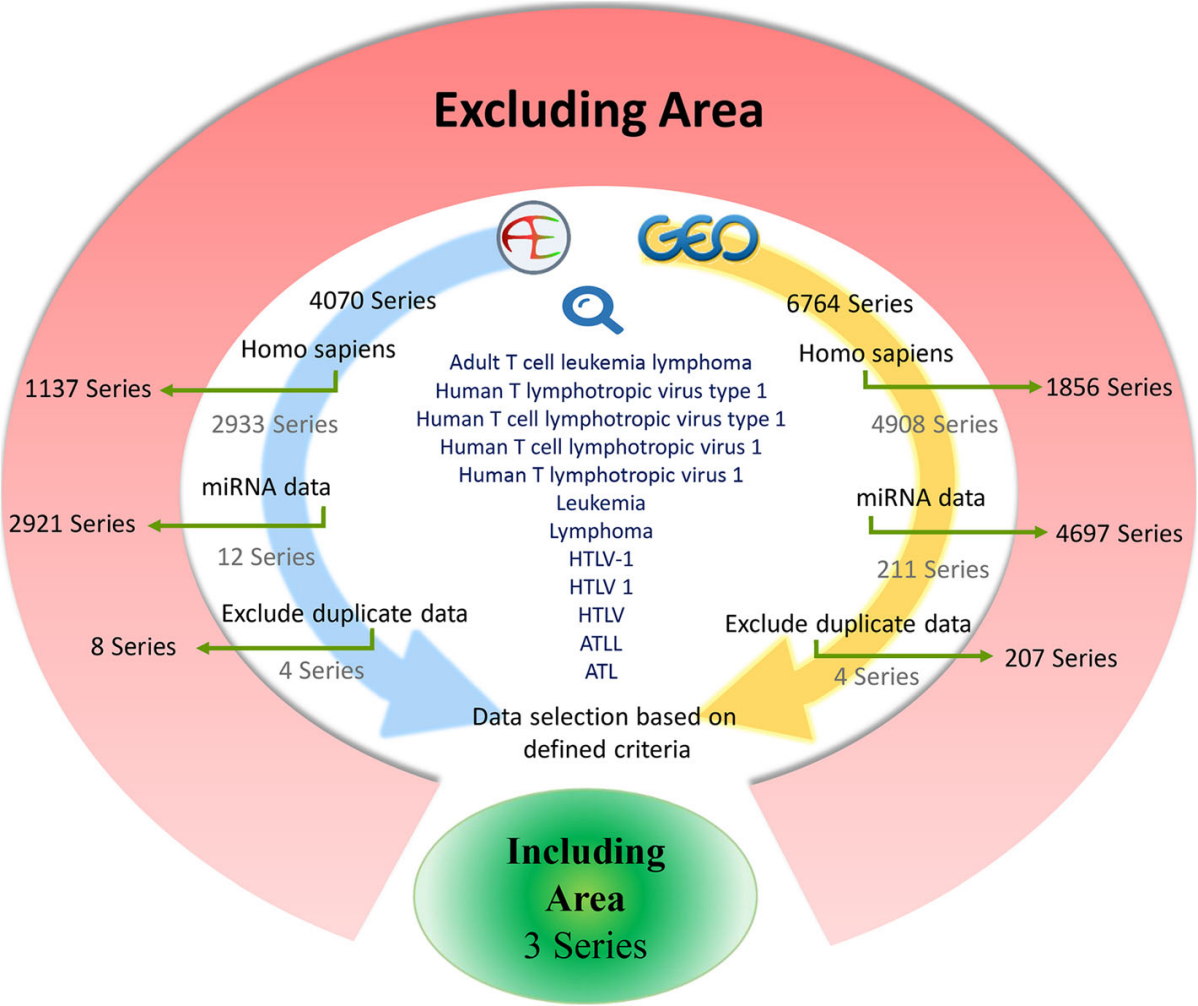
Search strategy and exclusion criteria of the study. Out of 6764 entries in Gene Expression Omnibus (GEO) and 4070 entries in ArrayExpress, 8 datasets remained after applying the exclusion criteria. Subsequently, 3 datasets fit the inclusion criteria, which were included in this study

We searched the public domains, Gene Expression Omnibus (https://www.ncbi.nlm.nih.gov/geo) and Array Express (https://www.ebi.ac.uk/arrayexpress) by the end of 2018 to find datasets relevant to the expression levels of miRNA in ATLL patients and healthy donors. Fig. 1 highlights the keywords used and the overall flowchart in data gathering. The inclusion criterion was research studies with human miRNA microarray datasets and samples derived from peripheral blood of ATLL patients and healthy donors. Duplicate results and studies in which the donors were receiving treatment for ATLL and those with healthy HTLV-1 carriers as controls were excluded.

### Pre-processing and differential expression analysis

The selected datasets were pre-processed using GEOquerry package implemented in R programming language (Version 4.0.3). The datasets were normalized using log2 transformation in Affy package and were then integrated with MetaDE package in R programming language to identify DEMs. The proportion of DEMs with a p-value of less than 0.001 were considered for further analysis. Subsequently, the gene targets for selected DEMs were determined by miRDB online database (http://mirdb.org/miRDB/) [27]. The associated genes were included with a target score of larger than 20.

### Network construction and pathway enrichment analysis

The STRING database version 11.0 was employed to construct protein-protein interactions network (PPIN) for gene targets of each DEMs based on literature sources for protein-protein interactions. The interactions included for network construction in this study include physical and functional interactions, high-throughput experiments, co-expression, genomic context, databases, and text-mining. The primary PPINs analysis was conducted using NetworkAnalyzer in Cytoscape 3.5.1 [28]. Genes with higher *degree* and *betweenness* centrality measures were computed to determine hub genes. Lastly, the PPINs were reconstructed and visualized using Gephi (0.9.1) based on calculated gene hubs.

Gene enrichment analysis was carried out to enrich hub genes in each DEMs in EnrichR web tool using Kyoto Encyclopedia of Genes and Genomes (KEGG) pathways [29, 30]. A combined score of higher than 0.4 was considered as cut-off to analyze the PPINs.

### Patient population and sample collection

The blood samples were collected from 8 ATLL patients and 10 healthy subjects between 2019 and 2020 from Shariati Hospital, Tehran, Iran. All samples were collected after acquiring informed consent from the patients or their next of kin when appropriate. A standardized clinical checklist, comprising demographic information and the diagnosis of ATLL was evaluated by a trained hematologist. None of the included patients were receiving chemotherapy and/or anti-cancer drugs. All methods were carried out in accordance with the relevant guidelines and regulations. The enzyme-linked immunosorbent assay (ELISA, Diapro, Italy) was used to perform the serology test for HTLV-1. PCR was then employed to confirm the serology results [31]. The inclusion criteria for healthy donors were participants with no active acute infectious disease, no concurrent drug use, and no diagnosed genetic abnormalities or defects. This investigation was approved by the Ethics Committee of Biomedical Research at Alborz University of Medical Sciences (IR.NIMAD.REC.1397.473).

### Quantitative reverse transcriptase PCR and statistical analysis

Total RNA was extracted from fresh whole blood utilizing TriPure isolation reagent (Roche, Germany). cDNA was synthesized using RT-ROSET Kit (ROJETechnologies, Iran) and SYBR Green-based (TaKaRa, Otsu, Japan) and subsequently, RT-qPCR was performed, according to the manufacturers’ instructions. The following primers were utilized to determine the expression levels of JNK, EVI1, MKP, and PTPRR and to confirm HTLV-1 infected cells in the samples: EVI1 (forward primer (FP): 5′-TCGTCGCCTCATTCTGAACTGGAA-3′, reverse primer (RP): 5′-ACTGCCATTCATTCTCTCCTCCACA-3′) MKP (FP: 5′-AGCCACCATCTGCCTTGCT-3′ , RP: 5′-CCAGCCTCTGCCGAACAGT-3′ ) PTPPR (FP: 5′-CCAGCACTGTCCGAGGCAA-3′ , RP: 5′-GCAAACAGAGGTAGCGGTGGT-3′ ) JNK (FP: 5′-TGCTGTGTGGAATCAAGCACCT-3′ , RP: 5′-TCGGGTGCTCTGTAGTAGCGA-3′ ) HBZ (FP: 5′-ACGTCGCCCGGAGAAAACA-3′ , RP: 5′-CTCCACCTCGCCTTCCAACT-3′) 5’LTR (FP: 5′-GGCTCGCATCTCCCCTTCAC-3′ , RP: GAGCAAGCAGGGTCAGGCAA-3′). The relative two standard curves real-time PCR was performed on the cDNA samples using Q-6000 machine (Qiagen, Germany). The GAPDH gene was utilized to normalize the mRNA expression levels respectively, as well as to control the error between samples [32]. The output for each group was analyzed using Mann-Whitney U test for statistical difference between gene expression. A *p*-value of less than 0.05 was considered to be significant.

## Results

After removal of redundancy, application of inclusion and exclusion criteria, and quality control using MetaQC package in R, 3 datasets namely, GSE28626 [33], GSE31629 [34], and GSE11577 [35] were selected for the DEM analysis of ATLL patients vs healthy individuals.

The primary analysis of microarray datasets identified hsa-let-7a, hsa-let-7g, hsa-mir-181b, hsa-mir-26b, hsa-mir-30c, hsa-mir-186, hsa-mir-10a, hsa-mir-30b, and hsa-let-7f as DEMs in ATLL patients compared to normal individuals. The target genes for each mentioned DEM were identified using miRDB. The analysis of networks using centrality parameters was utilized to select nodes with higher degree and betweenness as hub genes.

### Network analysis and PPIN characteristics

**Fig. 2:**
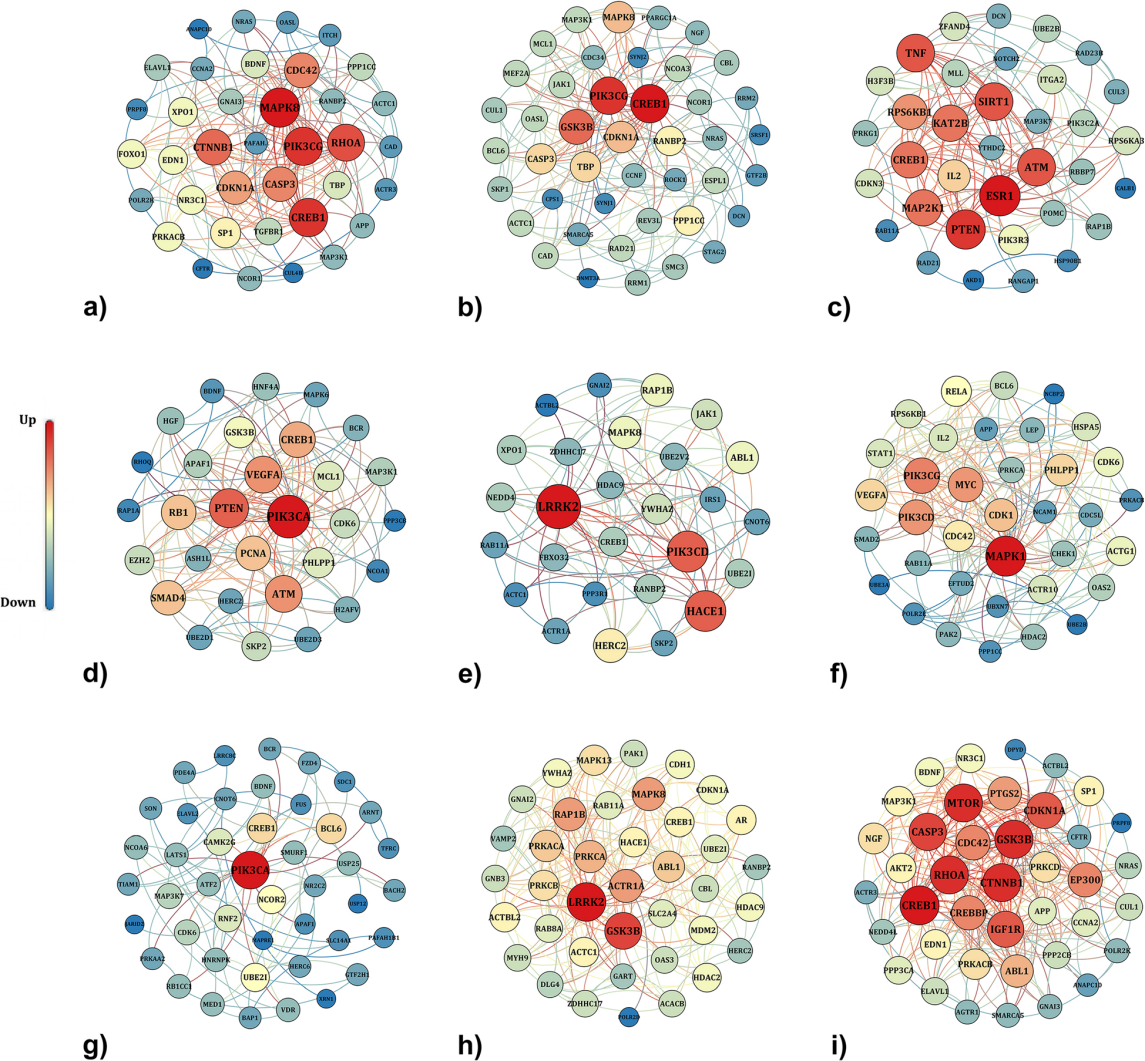
Protein-protein interaction networks of the enriched hub genes of differentially expressed miRNAs. PPINs of the identified target genes of **a** hsa-let-7a, **b** hsa-let-7g, **c** hsa-mir-181b, **d** hsa-mir-26b, **e** hsa-mir-30c, **f** hsa-mir-186, **g** hsa-mir-10a, **h** hsa-mir-30b, and **i** hsa-let-7f is illustrated. Genes with higher degree and betweenness are demonstrated in the center in red

The PPINs were constructed for each DEM separately using STRING to highlight the relationship between the target genes (Fig. 2). The networks were comprised of (a) 37 nodes and 176 edges for gene targets of hsa-let-7a, (b) 43 nodes and 167 edges for gene targets of hsa-let-7g, (c) 35 nodes and 125 edges for gene targets of hsa-mir-181b, (d) 30 nodes and 149 edges for gene targets of hsa-mir-26b, (e) 27 nodes and 98 edges for gene targets of hsa-mir-30c, (f) 36 nodes and 191 edges for gene targets of hsa-mir-186, (g) 44 nodes and 82 genes for gene targets of hsa-mir-10a, (h) 39 nodes and 217 edges for gene targets of hsa-mir-30b, and (i) 40 nodes and 257 edges for gene targets of hsa-let-7f.

### GO/Pathway enrichment analysis of identified genes

The Hub genes of all defined DEMs were enriched to reveal biological pathways associated with the PPINs. The analysis highlighted the following pathways for each DEM: hsa-let-7a: Endometrial cancer, Colorectal cancer, Renin secretion, Cocaine addiction, TNF signaling pathway, Human T-cell leukemia virus 1 infection, Thyroid hormone signaling pathway, Human cytomegalovirus infection, Renal cell carcinoma, Cortisol synthesis and secretion.

hsa-let-7g: Endometrial cancer, Colorectal cancer, Human T-cell leukemia virus 1 infection, Circadian rhythm, Thyroid hormone signaling pathway, Toxoplasmosis, TNF signaling pathway, Hedgehog signaling pathway, Human cytomegalovirus infection, Chronic myeloid leukemia;

hsa-mir-181b: Endocrine and other factor-regulated calcium reabsorption, Endometrial cancer, TNF signaling pathway, Renal cell carcinoma, Acute myeloid leukemia, Thyroid hormone signaling pathway, Human T-cell leukemia virus 1 infection, Toll-like receptor signaling pathway, Small cell lung cancer, Graft-versus-host disease.

hsa-mir-26b: Endometrial cancer, Small cell lung cancer, Renal cell carcinoma, Melanoma, Amyotrophic lateral sclerosis (ALS), Chronic myeloid leukemia, Human T-cell leukemia virus 1 infection, Renin secretion, Cocaine addiction, Colorectal cancer.

hsa-mir-30c: Renin secretion, Endocrine and other factor-regulated calcium reabsorption, TNF signaling pathway, Osteoclast differentiation, Endometrial cancer, Th17 cell differentiation, Human T-cell leukemia virus 1 infection, Cocaine addiction, Aldosterone-regulated sodium reabsorption, Renal cell carcinoma.

hsa-mir-186: Endocrine and other factor-regulated calcium reabsorption, Endometrial cancer, Renal cell carcinoma, Acute myeloid leukemia, Thyroid hormone signaling pathway, Salmonellosis, Chronic myeloid leukemia, Pancreatic cancer, Colorectal cancer, Th17 cell differentiation.

hsa-mir-10a: TNF signaling pathway, Cocaine addiction, Circadian rhythm, Small cell lung cancer, Endocrine and other factor-regulated calcium reabsorption, Endometrial cancer, Renal cell carcinoma, Chronic myeloid leukemia, Cortisol synthesis and secretion, Parathyroid hormone synthesis, secretion and action.

hsa-mir-30b: Endocrine and other factor-regulated calcium reabsorption, Endometrial cancer, Cocaine addiction, Thyroid hormone signaling pathway, Chronic myeloid leukemia, Human cytomegalovirus infection, Long-term depression, Salivary secretion, Renin secretion, Salmonella infection.

hsa-let-7f: Endometrial cancer, Renin secretion, Renal cell carcinoma, Colorectal cancer, Human cytomegalovirus infection, Thyroid hormone signaling pathway, Long-term depression, Cocaine addiction, Prostate cancer, Neurotrophin signaling pathway. The following five DEMs were directly associated with HTLV-1 related pathogenic pathways: hsa-let-7a, hsa-let-7g, hsa-mir-181b, hsa-mir-26b, hsa-mir-30c (Table 1).

**Table 1:** The significant biological pathways enriched by hub genes of DEMs

Row	miRNA	Enriched terms
1	hsa-let-7a	Endometrial cancer, Colorectal cancer, Renin secretion, Cocaine addiction, TNF signaling pathway, Human T-cell leukemia virus 1 infection, Thyroid hormone signaling pathway, Human cytomegalovirus infection, Renal cell carcinoma, Cortisol synthesis and secretion
2	hsa-let-7g	Endometrial cancer, Colorectal cancer, Human T-cell leukemia virus 1 infection, Circadian rhythm, Thyroid hormone signaling pathway, Toxoplasmosis, TNF signaling pathway, Hedgehog signaling pathway, Human cytomegalovirus infection, Chronic myeloid leukemia
3	hsa-mir-181b	Endocrine and other factor-regulated calcium reabsorption, Endometrial cancer, TNF signaling pathway, Renal cell carcinoma, Acute myeloid leukemia, Thyroid hormone signaling pathway, Human T-cell leukemia virus 1 infection, Toll-like receptor signaling pathway, Small cell lung cancer, Graft-versus-host disease
4	hsa-mir-26b	Endometrial cancer, Small cell lung cancer, Renal cell carcinoma, Melanoma, Amyotrophic lateral sclerosis (ALS), Chronic myeloid leukemia, Human T-cell leukemia virus 1 infection, Renin secretion, Cocaine addiction, Colorectal cancer
5	hsa-mir-30c	Renin secretion, Endocrine and other factor-regulated calcium reabsorption, TNF signaling pathway, Osteoclast differentiation, Endometrial cancer, Th17 cell differentiation, Human T-cell leukemia virus 1 infection, Cocaine addiction, Aldosterone-regulated sodium reabsorption, Renal cell carcinoma
6	hsa-mir-186	Endocrine and other factor-regulated calcium reabsorption, Endometrial cancer, Renal cell carcinoma, Acute myeloid leukemia, Thyroid hormone signaling pathway, Salmonella infection, Chronic myeloid leukemia, Pancreatic cancer, Colorectal cancer, Th17 cell differentiation
7	hsa-mir-10a	TNF signaling pathway, Cocaine addiction, Circadian rhythm, Small cell lung cancer, Endocrine and other factor-regulated calcium reabsorption, Endometrial cancer, Renal cell carcinoma, Chronic myeloid leukemia, Cortisol synthesis and secretion, Parathyroid hormone synthesis, secretion and action
8	hsa-mir-30b	Endocrine and other factor-regulated calcium reabsorption, Endometrial cancer, Cocaine addiction, Thyroid hormone signaling pathway, Chronic myeloid leukemia, Human cytomegalovirus infection, Long-term depression, Salivary secretion, Renin secretion, Salmonella infection
9	hsa-let-7f	Endometrial cancer, Renin secretion, Renal cell carcinoma, Colorectal cancer, Human cytomegalovirus infection, Thyroid hormone signaling pathway, Long-term depression, Cocaine addiction, Prostate cancer, Neurotrophin signaling pathway

Manual approach to enrich target genes with higher network connectivity also demonstrated the association of hub genes with several biological and signaling pathways including cell cycle regulation and DNA damage response (CDKN1A, RB1, SKP1, CDK6, SKP1, SKP2, CUL1, CDK1, ATM , SMC, XPO1, UBE2D1, RANBP2, ACTR1A, ESPL1, RANGAP1, ANAPC10, PPP1CC, PRKACA, RAD21, PAFAH1B1, ABL1, RHOA, TNF, RRM2, MTOR, APP, PAK1, CHEK1), MAP kinase (MAP 2K1, MAP3K1, MAPK8, MEF2A, MAP3K7, NRAS, MYC, YWHAZ, IL2, NCAM1, CUL3, LRRK2, PAK1) Phosphatidylinositol-3-kinase (PIK3CG, PIK3C2A, EDN1, PIK3CA, PIK3CD, IGF1R, PTEN) pathways among others.

### Validation by qRT-PCR assay

**Fig. 3:**
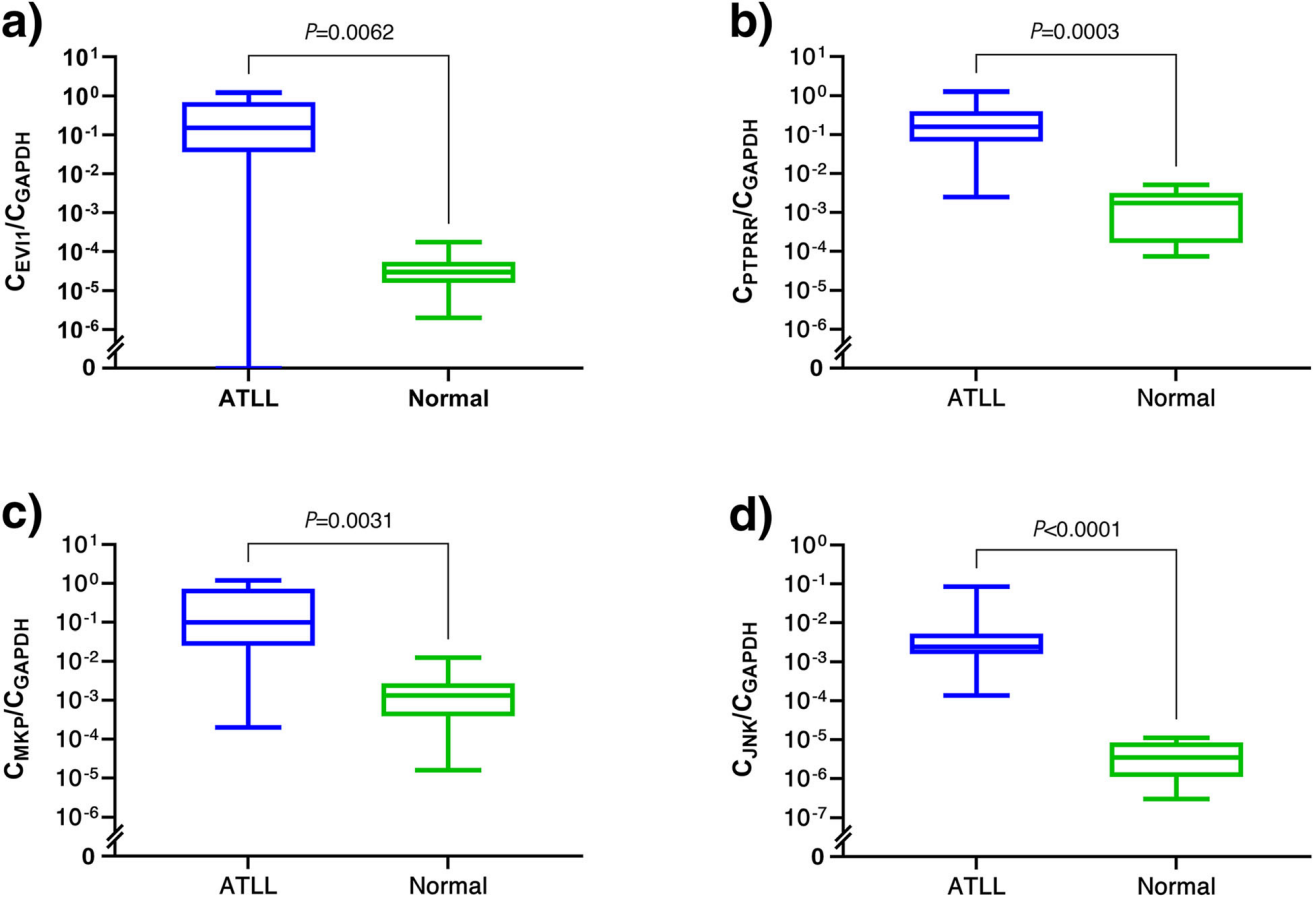
Gene expression of MAPK8 (JNK) and its major inhibitors in the MAPK/JNK signaling pathway. Quantitative RT-PCR revealed significant upregulation of JNK (*p-value*<0.0001) and its major inhibitors, EVI1 (*p-value* = 0.0062), PTPRR (*p-value* = 0.0003), and MKP1 (*p-value* = 0.0031). Error bars represent minimum and maximum data points in each assay. A single outlier sample with 0 value was observed in ATLL group for EVI1 PCR assay, removal of which did not affect the significance of the differential expression between the groups

The prominent involvement of top common enriched hub genes (Table 2) in MAP kinase signaling cascade, particularly in the JNK pathway, had led us to analyze the gene expression of JNK (MAPK8) and its major regulators, namely, ecotropic viral integration site 1 (EVI1), Dual-specificity phosphatase-1 (DUSP 1/MKP), and Protein tyrosine phosphatase receptor-type R (PTPRR) in ATLL patients and healthy subjects to validate the results of the meta-analysis. The results demonstrated significant upregulation of *EVI1* (*p*-value = 0.0062), *MKP* (*p*-value = 0.0003), *PTPRR* (*p*-value = 0.0031), and JNK (*p*-value < 0.0001) in ATLL patients compared to healthy controls (Fig. 3).

**Table 2:** Common hub genes among DEMs

Row	miRNA name	Common hub genes
1	hsa-let-7a, hsa-let-7g, hsa-mir-181b, hsa-mir-26b, hsa-mir-30c	CREB1
2	hsa-let-7a, hsa-let-7g, hsa-mir-26b	MAP3K1
3	hsa-let-7a, hsa-let-7g, HSA-mir-30c	MAPK8, RANBP2, ACTC1
4	hsa-let-7g, hsa-mir-26b	GSK3B, MCL1
5	hsa-let-7g, hsa-mir-30c	JAK1
6	hsa-let-7g, hsa-mir-181b	DCN, RAD21
7	hsa-let-7a, hsa-let-7g	PPP1CC, CAD, TBP, CDKN1A, NCOR1, OASL, PIK3CG, NRAS (RAS), CASP3
8	hsa-mir-26b, hsa-mir-30c	SKP2, HERC2
9	hsa-mir-181b, hsa-mir-26b	PTEN, ATM
10	hsa-let-7a, hsa-mir-26b	BDNF
11	hsa-mir-181b, hsa-mir-30c	RAB11A, RAP1B
12	hsa-let-7a, hsa-mir-30c	XPO1

## Discussion

Molecular approaches have led to the identification of several DEMs and their aberrant expression in array studies. While having their own merits, the conditions of these experiments could direct to perturbations in the analysis of differential gene expression in studies restricted to limited samples, as seen in incongruity of previously reported ATLL gene expression studies [26, 36]. The high throughput analysis of previous datasets as an alternative method can solidify the results of previous studies while providing novel insights into the genetic profile of the diseases [32, 37]. Through analysis of differentially expressed miRNAs in three independent microarray datasets in this study, we highlighted 9 miRNAs associated with in peripheral blood cells of ATLL patients. Further analysis revealed more than 300 target genes for the DEMs in the literature, emphasizing the vast disruption of biological processes in ATLL.

The identified DEMs in this study are linked to several neoplasms and their progression and subsequent metastasis. Hsa-let-7a, has-let-7g, hsa-mir-181b, hsa-miR-26b have been observed to act as tumor suppressors in certain malignancies by reducing the levels of c-myc oncogene which is overexpressed in at least 40% of human cancers [38–42]. Additionally, has-let-7f also inhibits gastric cancer invasion and metastasis through interaction with MYH9 mRNA [43]. Contrarily, has-miR-30c expression increases the invasiveness of metastatic neoplastic cells and is associated with poor prognosis in breast cancer by inhibiting NOV/CCN3 regulatory proteins [44]. The dysregulation of has-miR-10a is also observed in multiple malignancies, with high expression levels in urothelial and medullary thyroid carcinoma and low expression values in chronic myeloid leukemia [45–47]. Moreover, miR-186-5p regulates IGF-1 expression and apoptosis in neuroblastoma cells and is also proposed as a screening biomarker in colorectal polyps and adenomas [48]. Lastly, has-let-7a-5p, hsa-miR-181b-5p, hsa-miR-26b-5p, has-miR-30c-5p were also demonstrated to be differentially expressed small RNAs in a recent study on ATLL [49].

An interesting observation in this study was the extensive upregulation of JNK apoptotic pathway in the analysis, confirmed by upregulation of MAPK8 in ATLL samples. The implication of this finding is emphasized in the context of constant activation of NF-KB pathway observed in almost all ATLL clones that normally represses the JNK pathway [8, 50]. Furthermore, TNF-induced JNK activation, which is among upregulated DEGs in this study, normally results in apoptosis and cell death in target cells [51]. Therefore, to be able to explain this inconsistency, we examined the expression levels of the major inhibitors of JNK, namely, EVI1, PTPRR, and MKP. To our knowledge, this is the first study to demonstrate such upregulation of JNK repressors in ATLL. Dysregulation of PTPRR and MKP are implicated in the development and progression of various cancers owing to their ability to regulate both p38 and JNK MAPK pathways [52–55]. EVI1 has also been recognized as one of the most aggressive oncogenes associated with human leukemias such as acute myeloid leukemia. Aberrant expression of EVI1 leads to repression of TGF-β signaling, upregulated cell proliferation, and impaired cellular differentiation [56]. The levels of EVI1 transcripts are also associated with a poor prognosis in serous epithelial ovarian cancer [57].

Counterintuitively, quantitative PCR assay revealed considerable upregulation of the analyzed JNK inhibitors. Previous studies have described upregulation of JNK pathway via Tax-mediated activation of TAK1 and MEKK1, constitutive activation of this pathway in HTLV-1 transformed cells, and their role in the virus-induced tumorigenesis [58–60]. Therefore, the high levels of expression of the JNK inhibitors coupled with general activation of the pathway demonstrates a complex disruption of JNK signaling cascade. Seemingly, the extensive stimulation of NF-KB pathway in ATLL interferes with the function of JNK through interaction with the various activators of JNK pathway including TNF signaling and downstream pathways leading to apoptosis [51, 61, 62]. This subverted JNK pathway is deprived of its pro-apoptotic activities and contributes to leukemogenesis by retaining its stimulation of AP1 and repression of p53 pathways which subsequently govern cell cycle and survival. Furthermore, this dysregulated pathway also promotes visceral invasion of ATLL leukemic cells by virtue of MMP-7 upregulation [63, 64]. Therapeutic targeting of JNK pathway thus proves to be an interesting topic for future studies.

## Conclusion

In this high throughput meta-analysis, we identified significant disruption of genes related to cell cycle, proliferation, and signal transduction in ATLL. Subsequent in vitro assay demonstrated higher gene expression of JNK (MAPK8) and the major regulators of MAPK/JNK in ATLL vs healthy controls. The results of this study provide further insight into the dysregulated biological processes in ATLL. Further studies are needed to identify novel and reliable biomarkers, and prognostic factors and to obtain a comprehensive mapping of deregulated biological pathways in ATLL.

## Data Availability

The datasets analyzed during this study are available in the gene expression omnibus public repository (www.ncbi.nlm.nih.gov/geo) [33–35].

## Abbreviations

HTLV-1: Human T lymphotropic virus 1
ATLL: Adult T-cell Leukemia/Lymphoma
miRNA: MicroRNA
HAM/TSP: HTLV-1-associated myelopathy/Tropical spastic paraparesis
PPIN: protein-protein interactions network
